# Exact-Factorization Framework for Electron–Nuclear Dynamics in Electromagnetic Fields

**DOI:** 10.1021/acs.jctc.5c02174

**Published:** 2026-04-15

**Authors:** Vladimir U. Nazarov, E. K. U. Gross

**Affiliations:** Fritz Haber Research Center for Molecular Dynamics and Institute of Chemistry, Hebrew University of Jerusalem, Jerusalem 9190401, Israel

## Abstract

The Exact Factorization (EF) theory aims at the separation of the nuclear and electronic degrees of freedom in the many-body (MB) quantum mechanical problem. Being formally equivalent to the solution of the MB Schrödinger equation, EF sets up a strategy for the construction of efficient approximations in the theory of the correlated electronic-nuclear motion. Here we extend the EF formalism to incorporate the case of a system under the action of electromagnetic fields. An important interplay between the physical magnetic and the Berry curvature fields is revealed and discussed within the fully nonadiabatic theory. In particular, it is a previously known property of the Born–Oppenheimer (BO) approximation that, for a neutral atom in a uniform magnetic field, the latter is compensated by the Berry curvature field in the nuclear equation of motion (Yin and Mead, *Theoret. Chim. Acta* **1992,** *82,* 397). From an intuitive argument that the atom must not be deflected by the Lorentz force from a straight line trajectory, it has been conjectured that the same compensation should occur within the EF theory as well. We prove that this property persists within the exact nonadiabatic theory, provided the atom is in its eigenstate. The latter finds itself in the conspicuous variance with the corresponding BO result, where the compensation holds for an arbitrary nuclear wave packet on a single BO potential surface.

## Graphical abstract

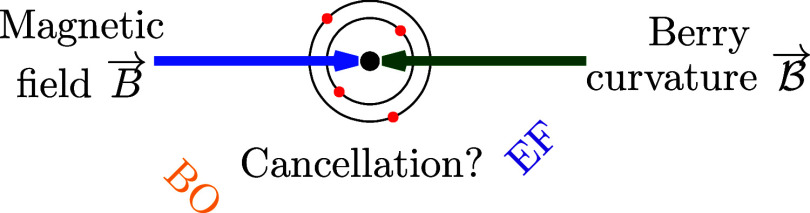

## 1 Introduction: The Concept of Exact Forces on the Nuclei

Consider a system of *N*_*e*_ electrons and *N*_*n*_ nuclei. In the absence of external fields, its propagation is described by the time-dependent Schrödinger equation

1 $$i\hbar \partial_{t}\Psi \left(\underset{=}{r},\underset{=}{R},t\right)=Ĥ\left(\underset{=}{r},\underset{=}{R}\right)\Psi \left(\underset{=}{r},\underset{=}{R},t\right)$$

where $\underset{=}{r}=\left(r_{1},r_{2},...r_{N_{e}}\right)$ and $\underset{=}{R}=\left(R_{1},R_{2},...R_{N_{n}}\right)$ represent the sets of electronic and nuclear position vectors. The Hamiltonian *Ĥ* contains the kinetic energies of electrons and nuclei and the mutual Coulomb interactions between all particles of the system

2 $$Ĥ={\mathrm{T̂}}_{n}+{Ĥ}^{BO}$$

where the Born–Oppenheimer (BO) Hamiltonian is defined as

$${Ĥ}^{BO}={\mathrm{T̂}}_{e}\left(\underset{=}{r}\right)+{Ĥ}_{ee}\left(\underset{=}{r}\right)+{Ĥ}_{en}\left(\underset{=}{r},\underset{=}{R}\right)+{Ĥ}_{nn}\left(\underset{=}{R}\right)$$

and

$$\begin{array}{l} {\mathrm{T̂}}_{n}=\sum_{I=1}^{N_{n}}-\frac{\hbar^{2}\nabla_{I}^{2}}{2M_{I}}\\ {\mathrm{T̂}}_{e}=\sum_{i=1}^{N_{e}}-\frac{\hbar^{2}\nabla_{i}^{2}}{2m}\\ {Ĥ}_{ee}=\sum_{i>j=1}^{N_{e}}\frac{e^{2}}{\left|r_{i}-r_{j}\right|}\\ {Ĥ}_{ne}=-\sum_{I=1}^{N_{n}}\sum_{i=1}^{N_{e}}\frac{e^{2}Z_{I}}{\left|r_{i}-R_{I}\right|}\\ {Ĥ}_{nn}=\sum_{I>J=1}^{N_{n}}\frac{e^{2}Z_{I}Z_{J}}{\left|R_{I}-R_{J}\right|} \end{array}$$

*M*_*I*_ and *eZ*_*I*_ denote the mass and charge of the *I*-th nucleus, *m* and −*e* are the mass and charge of electron. Let us now focus on one single nucleus with index *I* and ask what the force on this particular nucleus is. The answer is clear: Following the steps first formulated by Ehrenfest in the early days of quantum mechanics,^1^ we determine the momentum expectation value of this particular nucleus from the full electron–nuclear wave function, i.e., the solution of eq 1

3 $$P_{I}\left(t\right)={\left\langle \Psi \left(t\right)\left|-i\hbar \nabla_{R_{I}}\right|\Psi \left(t\right)\right\rangle }_{\underset{=}{r}\underset{=}{R}}$$

Next we evaluate the time derivative of the momentum expectation value eq 3 yielding

$$\frac{dP_{I}\left(t\right)}{dt}={\left\langle \Psi \left(t\right)\left|{\mathrm{f̂}}_{I}\right|\Psi \left(t\right)\right\rangle }_{\underset{=}{r}\underset{=}{R}}$$

The resulting force operator ${\mathrm{f̂}}_{I}$ obviously consists of the sum of all bare Coulomb forces exerted on the *I*-th nucleus by the electrons and by all other nuclei, i.e.

4 $${\mathrm{f̂}}_{I}=-\nabla_{R_{I}}\left({Ĥ}_{ne}+{Ĥ}_{nn}\right)$$

While mathematically doubtlessly correct, eq 4 is not a very useful way of writing the force on a given nucleus because it still involves the electronic degrees of freedom explicitly. Moreover, eq 4 obscures the fact that, for most systems, the low-lying modes of the nuclear subsystem are collective vibrations implying that the forces should be of harmonic nature. Usually, when approximately evaluating the force on a given nucleus, we take the derivative of a BO surface. In this way, the electronic degrees of freedom are already taken care of but we make an approximation, the BO approximation. This raises the question, can one define an exact force on the *I*-th nucleus with the electronic degrees of freedom integrated out, but without making the BO or any other approximation? This is indeed possible with an approach known as the exact factorization^2,3^ (EF) where the solution of eq 1 is written in the form of a product

5 $$\Psi \left(\underset{=}{r},\underset{=}{R},t\right)=\chi \left(\underset{=}{R},t\right)\Phi_{\underset{=}{R}}\left(\underset{=}{r},t\right)$$

The physical meaning of the two factors becomes clear when looking at the absolute square of eq 5. The left-hand side, $|\Psi \left(\underset{=}{r},\underset{=}{R},t\right){|}^{2}$ , is the joint probability density of finding the nuclei at $\underset{=}{R}$ and the electrons at $\underset{=}{r}$ (at time *t*). Knowing that any joint probability can be written as a product of a marginal probability and a conditional probability, $|\chi \left(\underset{=}{R},t\right){|}^{2}$ is the (marginal) probability density of finding the nuclei at positions $\underset{=}{R}$ , while $|\Psi_{\underset{=}{R}}\left(\underset{=}{r},t\right){|}^{2}$ is the conditional probability density of finding the electrons at $\underset{=}{r}$ , given the nuclei are at $\underset{=}{R}$ . To be interpretable as a conditional probability, the electronic factor must satisfy the partial normalization condition

6 $$\int d^{3N_{e}}r|\Phi_{\underset{=}{R}}\left(\underset{=}{r},t\right){|}^{2}=1,\;\mathrm{for}\;each\underset{=}{\;R}$$

The equations of motion for the two wave functions χ and Φ can be deduced either from the McLachlan’s variational principle,^4^ or by the direct insertion of eq 5 into the Schrödinger eq 1 and making use of eq 6. χ and Φ are unique up to within a phase factor $\exp\left[i\theta \left(\underset{=}{R},t\right)\right]$ , where $\theta \left(\underset{=}{R},t\right)$ is real: Clearly the transformation

7 $$\begin{array}{l} \mathrm{\chi ̃}\left(\underset{=}{R},t\right)=\exp\left[i\theta \left(\underset{=}{R},t\right)\right]\chi \left(\underset{=}{R},t\right)\\ {\mathrm{\Phi ̃}}_{\underset{=}{R}}\left(\underset{=}{r},t\right)=\exp\left[-i\theta \left(\underset{=}{R},t\right)\right]\Phi_{\underset{=}{R}}\left(\underset{=}{r},t\right) \end{array}$$

leaves the full wave function (eq 5) unchanged and does not affect the partial normalization (eq 6). This freedom corresponds to a gauge transformation of the equations of motion.^3^ The equation of motion for $\chi \left(\underset{=}{R},t\right)$ is a standard *N*_*n*_-body Schrödinger equation which contains a time-dependent scalar potential given by

8 $$\epsilon \left(\underset{=}{R},t\right)={\left\langle \Phi_{\underset{=}{R}}\left(t\right)\left|{Ĥ}^{BO}-i\hbar \partial_{t}\right|\Phi_{\underset{=}{R}}\left(t\right)\right\rangle }_{\underset{=}{r}}+Q\left(\underset{=}{R},t\right)$$

where

9 $$Q\left(\underset{=}{R},t\right)=\sum_{I=1}^{N_{n}}\frac{1}{2M_{I}}\left[{\left\langle \hbar \nabla_{R_{I}}\Phi_{\underset{=}{R}}\left(t\right)|\hbar \nabla_{R_{I}}\Phi_{\underset{=}{R}}\left(t\right)\right\rangle }_{\underset{=}{r}}-A_{I}^{2}\left(\underset{=}{R},t\right)\right]$$

as well as a vector potential that has the form of a Berry connection

10 $$A_{I}\left(\underset{=}{R},t\right)={\left\langle \Phi_{\underset{=}{R}}\left(t\right)\left|-i\hbar \nabla_{R_{I}}\right|\Phi_{\underset{=}{R}}\left(t\right)\right\rangle }_{\underset{=}{r}}$$

The EF allows one to deduce an alternative representation of the exact force on nucleus *I*: One first inserts eqs 5 into [Disp-formula eq3], yielding

11 $$P_{I}\left(t\right)={\left\langle \chi \left(t\right)\left|-i\hbar \nabla_{R_{I}}+A_{I}\left(t\right)\right|\chi \left(t\right)\right\rangle }_{\underset{=}{R}}$$

This representation of the exact momentum expectation value is then used to evaluate the rate of change of the momentum **P**_*I*_(*t*)­

$$\frac{dP_{I}\left(t\right)}{dt}={\left\langle \chi \left(t\right)\left|{\mathrm{F̂}}_{I}\right|\chi \left(t\right)\right\rangle }_{\underset{=}{R}}$$

This equation allows one to identify the force operator as follows^5^

12 $${\mathrm{F̂}}_{I}={\mathrm{F̂}}_{I}+{\mathrm{D̂}}_{I}$$

13 $${\mathrm{F̂}}_{I}={Ê}_{I}+{\mathrm{B̂}}_{I}\times {\mathrm{v̂}}_{I}$$

with the electric-like and magnetic-like forces

14 $${Ê}_{I}=\partial_{t}{Â}_{I}-\nabla_{R_{I}}\epsilon$$

15 $${\mathrm{B̂}}_{I}=\nabla_{R_{I}}\times {Â}_{I}$$

and the velocity operator

16 $${\mathrm{v̂}}_{I}=\left(-i\hbar \nabla_{R_{I}}+{Â}_{I}\right)/M_{I}$$

Finally, the second term on the right-hand side of eq 12 is an internuclear Lorentz-like force, ${\mathrm{D̂}}_{I}$ , which acts on the *I*-th nucleus but involves the velocities of only the other nuclei

17 $${\mathrm{D̂}}_{G_{I}}=\sum_{J\neq I,G_{J}^{\prime }}\left(\partial_{G_{J}^{\prime }}A_{G_{I}}-\partial_{G_{I}}A_{G_{J}^{\prime }}\right){\mathrm{v̂}}_{G_{J}^{\prime }}$$

with *G*_*I*_ ∈ (*X*_*I*_, *Y*_*I*_, *Z*_*I*_). There is no classical analogue of eq 17. We emphasize that eqs 12–17 are the *exact* forces on the nuclei. Neither have we made a classical approximation,^6^ nor did we invoke the adiabatic approximation. A very interesting aspect is the appearance of the Lorentz-like forces in eqs 13 and 17. These forces have to be distinguished from their BO counterparts. The latter are associated with the adiabatic approximation

18 $$\Psi^{adiab}\left(\underset{=}{r},\underset{=}{R},t\right)=\chi^{adiab}\left(\underset{=}{R},t\right)\Phi_{\underset{=}{R}}^{BO}\left(\underset{=}{r}\right)$$

where a single BO state is multiplied with a nuclear wave packet $\chi^{adiab}\left(\underset{=}{R},t\right)$ . If the latter is determined by plugging eq 18 in the McLachlan variational principle, the variationally best nuclear wave function $\chi^{adiab}\left(\underset{=}{R},t\right)$ satisfies a Schrödinger equation which also contains a Berry connection-type vector potential. The latter is associated with a single BO state

19 $$A_{I}^{adiab}\left(\underset{=}{R}\right)={\left\langle \Phi_{\underset{=}{R}}^{BO}\left|-i\hbar \nabla_{R_{I}}\right|\Phi_{\underset{=}{R}}^{BO}\right\rangle }_{\underset{=}{r}}$$

Apart from its time-independence, the vector potential eq 19 can be very different from the exact vector potential eq 10. This is known from the fact that the geometric phases associated with eqs 10 and 19 are generally different.^7–9^ In other words, the adiabatic approximation eq 19 is not necessarily a good approximation for the exact vector potential eq 10.

In recent years, the concept of EF has led to ground breaking developments in molecular physics. It has, e.g., proven an effective method in handling the problem of the electronic decoherence, nuclear wave packet branching, and the role of conical intersections in the mixed nonadiabatic electronic-nuclear dynamics.^10–16^

In this article we will further investigate the role of the vector potential eq 10, extending the theory to the case of systems under the action of external electromagnetic fields. A particularly interesting situation appears when the Berry curvature, i.e., the magnetic-field-like object associated with the vector potential eq 10, competes with a genuine external magnetic field. The purpose of this article is 2-fold: First we generalize the EF formalism to include systems exposed to an arbitrary time-dependent external electromagnetic field. This is a valid goal in its own right, as it extends the power of the EF formalism to the realm of magnetic phenomena. Although the diabatic electron–nuclear dynamics in magnetic field has been recently studied with the approximation of the Phase-Space method,^17^ to the best of our knowledge, the same has not been done within the exact EF framework. Second, the combined effect of the external and the Berry curvature magnetic fields is shown to be of great consequence: We prove that, for an eigenstate of a neutral atom, the two fields compensate each other exactly in the nuclear Schrödinger equation, ensuring the atom’s free motion in the uniform magnetic field. The corresponding property has been known before to hold in BO dynamics without the requirement of the eigenstate^18,19^ and, on the basis of intuitive arguments, it had been anticipated to hold in the exact theory as well.^20^ This difference is shown to crucially distinguish the diabatic and adiabatic answers to the problem of the two fields compensation.

This paper is organized as follows. In Secion [Sec sec2] we present the EF formalism extended to include arbitrary electromagnetic fields. Sections 3 and 4 are devoted to the proof of the cancellation between the physical and the Berry curvature magnetic fields in the case of an eigenstate of a neutral atom. In Section 5 we reconsider the problem of the two fields compensation under the BO approximation, demonstrating the spurious nature of the relaxation of the conditions on the compensation within this approximation. In Section 6, we give explicit examples of wave-packets violating the property of the Berry curvature compensation by the physical magnetic field. Conclusions are collected in Section 7. More technical derivations are deferred to Appendices.

## 2 Exact Factorization Formalism in the Presence of External Electromagnetic Fields

In the presence of the external vector ***A*** (***r***, *t*) and scalar ϕ­(***r***, *t*) potentials, the Hamiltonian eq 2 modifies to

20 $$Ĥ\left(t\right)={Ĥ}_{n}\left(t\right)+{Ĥ}^{BO}\left(t\right)$$

where

21 $${Ĥ}^{BO}\left(t\right)={Ĥ}_{e}\left(t\right)+{Ĥ}_{ee}+{Ĥ}_{ne}+{Ĥ}_{nn}$$

22 $${Ĥ}_{n}\left(t\right)=\sum_{I=1}^{N_{n}}\frac{1}{2M_{I}}{\left[-i\hbar \nabla_{R_{I}}-\frac{Z_{I}e}{c}A\left(R_{I},t\right)\right]}^{2}+eZ_{I}\phi \left(R_{I},t\right)$$

23 $${Ĥ}_{e}\left(t\right)=\frac{1}{2m}\sum_{i=1}^{N_{e}}{\left[-i\hbar \nabla_{r_{i}}+\frac{e}{c}A\left(r_{i},t\right)\right]}^{2}-eZ_{I}\phi \left(r_{i},t\right)$$

and *c* is the speed of light (our treatment follows the nonrelativistic semiclassical scheme, where electrons and nuclei are coupled to the electromagnetic field, but their mutual interaction is purely electrostatic^21^).

Equations of motion for $\chi \left(\underset{=}{R},t\right)$ and $\Phi_{\underset{=}{R}}\left(\underset{=}{r},t\right)$ entering eq 5, the derivation of which we defer until the Appendix A, read as follows

24 $$i\hbar \partial_{t}\chi \left(\underset{=}{R},t\right)=\sum_{I=1}^{N_{n}}\frac{1}{2M_{I}}{\left[-i\hbar \nabla_{R_{I}}+A_{I}^{tot}\left(\underset{=}{R},t\right)\right]}^{2}\chi \left(\underset{=}{R},t\right)+\epsilon^{tot}\left(\underset{=}{R},t\right)\chi \left(\underset{=}{R},t\right)$$

25 $$\begin{array}{l} i\hbar \partial_{t}\Phi_{\underset{=}{R}}\left(\underset{=}{r},t\right)=\left[{Ĥ}_{BO}\left(t\right)-\epsilon \left(\underset{=}{R},t\right)\right]\Phi_{\underset{=}{R}}\left(\underset{=}{r},t\right)+\sum_{I=1}^{N_{n}}\frac{1}{2M_{I}}{\left[-i\hbar \nabla_{R_{I}}-A_{I}\left(\underset{=}{R},t\right)\right]}^{2}\Phi_{\underset{=}{R}}\left(\underset{=}{r},t\right)\\ -\sum_{I=1}^{N_{n}}\frac{1}{M_{I}}\left[\frac{i\hbar \nabla_{R_{I}}\chi \left(\underset{=}{R},t\right)}{\chi \left(\underset{=}{R},t\right)}-A_{I}^{tot}\left(\underset{=}{R},t\right)\right]\cdot \left[-i\hbar \nabla_{R_{I}}-A_{I}\left(\underset{=}{R},t\right)\right]\Phi_{\underset{=}{R}}\left(\underset{=}{r},t\right) \end{array}$$

In eqs 24 and 25, the total vector potential $A_{I}^{tot}\left(\underset{=}{R},t\right)$ is comprised of the Berry connection $A_{I}\left(\underset{=}{R},t\right)$ and the external ***A*** (***R***_*I*_, *t*) parts

26 $$A_{I}^{tot}\left(\underset{=}{R},t\right)=A_{I}\left(\underset{=}{R},t\right)-\frac{Z_{I}e}{c}A\left(R_{I},t\right)$$

where $A_{I}\left(\underset{=}{R},t\right)$ is defined by eq 10, and

$$\epsilon^{tot}\left(\underset{=}{R},t\right)=\epsilon \left(\underset{=}{R},t\right)+\sum_{I=1}^{N_{n}}eZ_{I}\phi \left(R_{I},t\right)$$

where $\epsilon \left(\underset{=}{R},t\right)$ is given by eq 8.

We note that: (I) In the absence of the external magnetic field [***A*** (***r***, *t*) = **0**], eqs 24 and 25 reduce, obviously, to their nonmagnetic analogues;^2^ (II) If the magnetic field is present, it enters the nuclear equation of motion eq 24 through $A_{I}^{tot}\left(\underset{=}{R},t\right)$ of eq 26, the latter comprised additively of both the physical and the Berry connection vector potentials; (III) In the electronic equation of motion eq 25, $A_{I}^{tot}\left(\underset{=}{R},t\right)$ and $A_{I}\left(\underset{=}{R},t\right)$ enter separately, which breaks the symmetry between the physical and the Berry connection vector potentials; (IV) Regarding the latter asymmetry we note that, although the Hamiltonian of the electronic equation of motion is non-Hermitian, it conserves the partial normalization condition eq 6. It can be verified directly that, with the inclusion of the external electromagnetic field, the presence of $A_{I}\left(\underset{=}{R},t\right)$ , rather than $A_{I}^{tot}\left(\underset{=}{R},t\right)$ , in the second term on RHS of eq 25 and in the second factor of the third term is essential to ensure the preservation of the latter property, while the remaining $A_{I}^{tot}\left(\underset{=}{R},t\right)$ does not violate it. Noteworthily, the last term on RHS of eq 25 is separately invariant under the gauge transformation. This is in contrast to the two other terms in this equation, where the transformation leads to a redistribution of the value between $A_{I}\left(\underset{=}{R},t\right)$ and $\epsilon \left(\underset{=}{R},t\right)$ and, consequently, between the first and the second terms.

The power of EF lies largely in its providing a way to evaluate expectation values of some of the operators related to nuclei only, such as nuclear density, current density, and forces on nuclei, with the use of the nuclear wave function $\chi \left(\underset{=}{R},t\right)$ only. Since, so far, this property has been known to hold for systems without magnetic field, below we prove it anew with inclusion of the latter.

### 2.1 Nuclear Density and Current Density

The density operator of the *I*-the nucleus is

$${\mathrm{N̂}}_{I}\left(R\right)=\delta \left(R_{I}-R\right)$$

Therefore, the expectation value of the density reads

$$N_{I}\left(R,t\right)={\left\langle \Psi \left(t\right)\left|\delta \left(R_{I}-R\right)\right|\Psi \left(t\right)\right\rangle }_{\underset{=}{r}\underset{=}{R}}$$

Making the substitution (eq 5), integrating over electronic coordinates $\underset{=}{r}$ , and using the condition (eq 6), we have trivially

$$N_{I}\left(R,t\right)={\left\langle \chi \left(t\right)\left|\delta \left(R_{I}-R\right)\right|\chi \left(t\right)\right\rangle }_{\underset{=}{R}}$$

Furthermore, the operator of the current-density of the *I*-th nucleus is

$$\begin{array}{l} {Ĵ}_{I}\left(R\right)=-\frac{i\hbar }{2M_{I}}\left[\nabla_{R_{I}}\delta \left(R_{I}-R\right)+\delta \left(R_{I}-R\right)\nabla_{R_{I}}\right]\\ -\frac{Z_{I}e}{M_{I}c}A\left(R_{I},t\right)\delta \left(R_{I}-R\right) \end{array}$$

Therefore

$$\begin{array}{l} J_{I}\left(R,t\right)=-\frac{i\hbar }{2M_{I}}\langle \chi \left(t\right)\Phi_{\underset{=}{R}}\left(t\right)|\nabla_{R_{I}}\delta \left(R_{I}-R\right)+\delta \left(R_{I}-R\right)\nabla_{R_{I}}|\chi \left(t\right)\Phi_{\underset{=}{R}}{\left(t\right)\rangle }_{\underset{=}{r}\underset{=}{R}}\\ -\frac{Z_{I}e}{M_{I}c}\langle \chi \left(t\right)\Phi_{\underset{=}{R}}\left(t\right)|A\left(R_{I},t\right)\delta \left(R_{I}-R\right)|\chi \left(t\right)\Phi_{\underset{=}{R}}{\left(t\right)\rangle }_{\underset{=}{r}\underset{=}{R}} \end{array}$$

or

$$\begin{array}{l} J_{I}\left(R,t\right)=\frac{i\hbar }{2M_{I}}{\left\langle \Phi_{\underset{=}{R}}\left(t\right)\left[\nabla_{R_{I}}\chi \left(t\right)\right]+\chi \left(t\right)\left[\nabla_{R_{I}}\Phi_{\underset{=}{R}}\left(t\right)\right]\left|\delta \left(R_{I}-R\right)\right|\chi \left(t\right)\Phi_{\underset{=}{R}}\left(t\right)\right\rangle }_{\underset{=}{r}\underset{=}{R}}\\ -\frac{i\hbar }{2M_{I}}{\left\langle \chi \left(t\right)\Phi_{\underset{=}{R}}\left(t\right)\left|\delta \left(R_{I}-R\right)\nabla_{R_{I}}\right|\Phi_{\underset{=}{R}}\left(t\right)\left[\nabla_{R_{I}}\chi \left(t\right)\right]+\chi \left(t\right)\left[\nabla_{R_{I}}\Phi_{\underset{=}{R}}\left(t\right)\right]\right\rangle }_{\underset{=}{r}\underset{=}{R}}\\ -\frac{Z_{I}e}{M_{I}c}{\left\langle \chi \left(t\right)\Phi_{\underset{=}{R}}\left(t\right)\left|A\left(R_{I},t\right)\delta \left(R_{I}-R\right)\right|\chi \left(t\right)\Phi_{\underset{=}{R}}\left(t\right)\right\rangle }_{\underset{=}{r}\underset{=}{R}} \end{array}$$

which, after the integration over $\underset{=}{r}$ and the use of the definition (10), reduces to

$$\begin{array}{l} J_{I}\left(R,t\right)=\frac{i\hbar }{2M_{I}}{\left\langle \Phi_{\underset{=}{R}}\left(t\right)\left[\nabla_{R_{I}}\chi \left(t\right)\right]\left|\delta \left(R_{I}-R\right)\right|\chi \left(t\right)\Phi_{\underset{=}{R}}\left(t\right)\right\rangle }_{\underset{=}{r}\underset{=}{R}}+\frac{i\hbar }{2M_{I}}{\left\langle \chi \left(t\right)\left[\nabla_{R_{I}}\Phi_{\underset{=}{R}}\left(t\right)\right]\left|\delta \left(R_{I}-R\right)\right|\chi \left(t\right)\Phi_{\underset{=}{R}}\left(t\right)\right\rangle }_{\underset{=}{r}\underset{=}{R}}\\ -\frac{i\hbar }{2M_{I}}{\left\langle \chi \left(t\right)\Phi_{\underset{=}{R}}\left(t\right)\left|\delta \left(R_{I}-R\right)\right|\Phi_{\underset{=}{R}}\left(t\right)\nabla_{R_{I}}\chi \left(t\right)\right\rangle }_{\underset{=}{r}\underset{=}{R}}-\frac{i\hbar }{2M_{I}}{\left\langle \chi \left(t\right)\Phi_{\underset{=}{R}}\left(t\right)\left|\delta \left(R_{I}-R\right)\right|\chi \left(t\right)\nabla_{R_{I}}\Phi_{\underset{=}{R}}\left(t\right)\right\rangle }_{\underset{=}{r}\underset{=}{R}}\\ -\frac{Z_{I}e}{M_{I}c}{\left\langle \chi \left(t\right)\Phi_{\underset{=}{R}}\left(t\right)\left|A\left(R_{I},t\right)\delta \left(R_{I}-R\right)\right|\chi \left(t\right)\Phi_{\underset{=}{R}}\left(t\right)\right\rangle }_{\underset{=}{r}\underset{=}{R}} \end{array}$$

Then

$$\begin{array}{l} J_{I}\left(R,t\right)=\frac{\hbar }{M_{I}}Im{\left\langle \chi \left(t\right)\left|\delta \left(R_{I}-R\right)\right|\nabla_{R_{I}}\chi \left(t\right)\right\rangle }_{\underset{=}{R}}\\ +\frac{1}{M_{I}}{\left\langle \chi \left(t\right)\left|A_{I}\left(t\right)\delta \left(R_{I}-R\right)\right|\chi \left(t\right)\right\rangle }_{\underset{=}{R}}-\frac{Z_{I}e}{M_{I}c}{\left\langle \chi \left(t\right)\left|A\left(t\right)\delta \left(R_{I}-R\right)\right|\chi \left(t\right)\right\rangle }_{\underset{=}{R}} \end{array}$$

Using eq 26, we finally have

27 $$J_{I}\left(R,t\right)=\frac{\hbar }{M_{I}}Im\langle \chi \left(t\right)|\delta \left(R_{I}-R\right)\left|\nabla_{R_{I}}\chi {\left(t\right)\rangle }_{\underset{=}{R}}+\langle \chi \left(t\right)|A_{I}^{tot}\left(t\right)\delta \left(R_{I}-R\right)\right|\chi {\left(t\right)\rangle }_{\underset{=}{R}}$$

### 2.2 Forces on Nuclei

In terms of the full many-body wave function, the momentum of the *I*-th nucleus is given by

$$P_{I}\left(t\right)={\left\langle \Psi \left(t\right)\left|-i\hbar \nabla_{R_{I}}-\frac{Z_{I}e}{c}A\left(R_{I},t\right)\right|\Psi \left(t\right)\right\rangle }_{\underset{=}{r}\underset{=}{R}}$$

Substituting the factorization (eq 5) in the last equation, we rewrite it readily as

28 $$P_{I}\left(t\right)={\left\langle \chi \left(t\right)\left|-i\hbar \nabla_{R_{I}}+A_{I}^{tot}\left(t\right)\right|\chi \left(t\right)\right\rangle }_{\underset{=}{R}}$$

where $A_{I}^{tot}\left(\underset{=}{R},t\right)$ is defined by eq 26. Comparing eq 28 with the corresponding eq 11 for the field-free case, we conclude that all the expressions for forces eqs 14–17 remain valid with the substitution of $A_{I}^{tot}\left(\underset{=}{R},t\right)$ for $A_{I}\left(\underset{=}{R},t\right)$ and $\epsilon^{tot}\left(\underset{=}{R},t\right)$ for $\epsilon \left(\underset{=}{R},t\right)$ . Namely

29 $${\mathrm{F̂}}_{I}={\mathrm{F̂}}_{I}+{\mathrm{D̂}}_{I}$$

30 $${\mathrm{F̂}}_{I}={Ê}_{I}+{\mathrm{B̂}}_{I}\times {\mathrm{v̂}}_{I}$$

31 $${Ê}_{I}=\partial_{t}{Â}_{I}^{tot}-\nabla_{R_{I}}\epsilon^{tot}$$

32 $${\mathrm{B̂}}_{I}=\nabla_{R_{I}}\times {Â}_{I}^{tot}$$

33 $${\mathrm{v̂}}_{I}=\left(-i\hbar \nabla_{R_{I}}+{Â}_{I}^{tot}\right)/M_{I}$$

34 $${\mathrm{D̂}}_{G_{I}}=\sum_{J\neq I,G_{J}^{\prime }}\left(\partial_{G_{J}^{\prime }}A_{G_{I}}^{tot}-\partial_{G_{I}}A_{G_{J}^{\prime }}^{tot}\right){\mathrm{v̂}}_{G_{J}^{\prime }}=\sum_{J\neq I,G_{J}^{\prime }}\left(\partial_{G_{J}^{\prime }}A_{G_{I}}-\partial_{G_{I}}A_{G_{J}^{\prime }}\right){\mathrm{v̂}}_{G_{J}^{\prime }}$$

As observables, forces must be invariant under two kinds of transformations, namely, the gauge freedom (eq 7) inherent to EF, and the usual electromagnetic gauge freedom associated with the vector and scalar potentials. To show that this holds explicitly, we first note that eq 34 is not affected by the electromagnetic potential ***A*** (***R***_*I*_, *t*), while its invariance under the transformation (eq 7) has been shown before.^5^ Furthermore, since the Berry connection and the electromagnetic vector potential enter eqs 31 and 32 additively, the invariance with respect to the two corresponding gauge transformations becomes evident. We note that the above check on the gauge invariance of eqs 29–34 is nothing else but a test of the faultlessness of their derivation from the laws of quantum mechanics, since (I) the covariance (invariance in form) of the Schrödinger equation with respect to the electromagnetic gauge transformation is a fundamental property and (II) EF method in its unabridged form is strictly equivalent to the solution of the MB quantum mechanical problem.

## 3 Neutral Atom in Uniform Magnetic Field

In this section we apply the EF method to the problem of a neutral atom’s motion in uniform magnetic field, proving that, under stationary conditions, the motion of the nucleus is that of a free particle. Since, in the case of a single nucleus, *D̂* of eq 17 is irrelevant, we will be concerned with the Berry curvature and the physical magnetic fields only.

In the case under consideration we have *N*_*n*_ = 1, *Z* = *N*_*e*_ = *N*, and ${Ĥ}_{nn}=0$ . We denote the coordinate of the nucleus by ***R*** and we use the gauge

35 $$A\left(r\right)=\frac{1}{2}B\times r$$

where ***B*** is the uniform magnetic field. The problem of the neutral atom in constant uniform magnetic field is known to conserve the pseudomomentum^22^

36 [Ĥ,K̂]=0

where

$$\mathrm{K̂}=-i\hbar \nabla_{R}+\frac{Ne}{c}A\left(R\right)+\sum_{i=1}^{N}\left[-i\hbar \nabla_{r_{i}}-\frac{e}{c}A\left(r_{i}\right)\right]$$

­(note the fundamental difference of signs at the vector potential as compared with the definition of the canonical momentum operator). Besides^22^

37 $$\left[{\mathrm{K̂}}_{i},{\mathrm{K̂}}_{j}\right]=0,,i,j=1,2,3$$

Introducing the coordinate of the center of mass (c.m.) and those of electrons relative to the nucleus

$$\begin{array}{l} R_{c}=\frac{MR+m\sum_{i=1}^{N}r_{i}}{M+Nm}\\ {\mathrm{r̃}}_{i}=r_{i}-R \end{array}$$

and, accordingly

$$\begin{array}{l} R=R_{c}-\frac{m}{M+Nm}\sum_{i=1}^{N}{\mathrm{r̃}}_{i}\\ r_{i}={\mathrm{r̃}}_{i}+R_{c}-\frac{m}{M+Nm}\sum_{i=1}^{N}{\mathrm{r̃}}_{i} \end{array}$$

and noting that

$$\begin{array}{l} \nabla_{R}=\frac{M}{M+Nm}\nabla_{R_{c}}-\sum_{i=1}^{N}\nabla_{{\mathrm{r̃}}_{i}}\\ \nabla_{r_{i}}=\frac{m}{M+Nm}\nabla_{R_{c}}+\nabla_{{\mathrm{r̃}}_{i}} \end{array}$$

we can write

38 $$\mathrm{K̂}=-i\hbar \nabla_{R_{c}}-\sum_{i=1}^{N}\frac{e}{2c}B\times {\mathrm{r̃}}_{i}$$

Due to the commutations eqs 36 and 37, the eigenfunction of the Hamiltonian can be chosen as also that of the pseudomomentum operator eq 38. Then

39 $$\left[-i\hbar \nabla_{R_{c}}-\sum_{i=1}^{N}\frac{e}{2c}B\times {\mathrm{r̃}}_{i}\right]\Psi \left(R_{c},\overset{\sim }{\underset{=}{r}}\right)=K\Psi \left(R_{c},\overset{\sim }{\underset{=}{r}}\right)$$

where **K** is an eigenvalue of **K̂**. Equation 39 is a differential equation with respect to ***R***_*c*_ only, where $\overset{\sim }{\underset{=}{r}}$ play the role of parameters. It can be readily solved, yielding

40 $$\Psi \left(R_{c},\overset{\sim }{\underset{=}{r}}\right)=\exp\left[\frac{i}{\hbar }\left(K+\frac{e}{2c}\sum_{i=1}^{N}B\times {\mathrm{r̃}}_{i}\right)\cdot R_{c}\right]\phi_{K}\left(\overset{\sim }{\underset{=}{r}}\right)$$

where the dependence of the eigenfunction of the relative motion $\phi_{K}\left(\overset{\sim }{\underset{=}{r}}\right)$ on **K**, inherent to the problem with magnetic field,^22,23^ is indicated by a subscript.

Taking use of eq 40 and returning to the particles’ individual coordinates, we write

$$\Psi \left(R,\underset{=}{r}\right)=\exp\left[\frac{i}{\hbar }\left(K+\frac{e}{2c}\sum_{i=1}^{N}B\times \left(r_{i}-R\right)\right)\cdot \frac{MR+m\sum_{i=1}^{N}r_{i}}{M+Nm}\right]\phi_{K}\left(\underset{=}{r}-R\right)$$

or

$$\Psi \left(R,\underset{=}{r}\right)=\exp\left[\frac{i}{\hbar }K\cdot \frac{MR+m\sum_{i=1}^{N}r_{i}}{M+Nm}\right]\exp\left[\frac{ie}{2\hbar c}\sum_{i=1}^{N}\left(B\times r_{i}\right)\cdot R\right]\phi_{K}\left(\underset{=}{r}-R\right)$$

In the EF context, we can choose the following splitting of $\Psi \left(R,\underset{=}{r}\right)$ into the χ­(***R***) and $\Phi_{R}\left(\underset{=}{r}\right)$ parts

41 $$\chi \left(R\right)=\exp\left(\frac{i}{\hbar }\frac{M}{M+Nm}K\cdot R\right)$$

42 $$\Phi_{R}\left(\underset{=}{r}\right)=\exp\left[\frac{i}{\hbar }\frac{m}{M+Nm}\sum_{i=1}^{N}K\cdot r_{i}\right]\exp\left[\frac{ie}{2\hbar c}\sum_{i=1}^{N}\left(B\times r_{i}\right)\cdot R\right]\phi_{K}\left(\underset{=}{r}-R\right)$$

Note that the partial normalization of $\Phi_{R}\left(\underset{=}{r}\right)$ eq 6 is ensured in eq 42 due to the normalization of $\phi_{K}\left(\overset{\sim }{\underset{=}{r}}\right)$ . All the splittings of $\Psi \left(R,\underset{=}{r}\right)$ other than according to eqs 41 and 42 would be equivalent to within the gauge transformation eq 7.

### Berry Connection A(R)

3.1

By the definition eq 10, we write

43 $$\begin{array}{l} A\left(R\right)=\frac{e}{2c}\langle \phi_{K}\left(\underset{=}{r}-R\right)|\sum_{i=1}^{N}\left(B\times r_{i}\right)|\phi_{K}{\left(\underset{=}{r}-R\right)\rangle }_{\underset{=}{r}}\\ -i\hbar \langle \phi_{K}\left(\underset{=}{r}-R\right)|\nabla_{R}|\phi_{K}{\left(\underset{=}{r}-R\right)\rangle }_{r} \end{array}$$

and then, due to the normalization of $\phi_{K}\left(\underset{=}{r}\right)$ to unity

44 $$A\left(R\right)=\frac{Ne}{2c}\left(B\times R\right)+A_{0}$$

where

45 $$A_{0}={\left\langle \phi_{K}\left(\underset{=}{r}\right)\left|\sum_{i=1}^{N}\frac{e}{2c}\left[B\times \left(r_{i}-R\right)\right]+i\hbar \nabla_{r_{i}}\right|\phi_{K}\left(\underset{=}{r}\right)\right\rangle }_{\underset{=}{r}}$$

is a constant vector. Therefore, according to eqs 26 and 35

46 $$A_{tot}\left(R\right)=A_{0}=const$$

which vector potential does not affect the motion of the nucleus, since it produces neither magnetic nor electric field.

### 3.2 Scalar Potential ϵ­(***R***)

Now we show that the scalar potential ϵ^tot^(***R***), entering eq 24, is constant as well, thus concluding the proof of the fact that the nucleus of a neutral atom in a uniform magnetic field moves as a free particle, provided the atom is in one of its eigenstates. Introducing the notation

$$\Pi \left(\underset{=}{r}\right)=\exp\left[\frac{im}{M+Nm}\sum_{i=1}^{N}K\cdot r_{i}\right]\phi_{K}\left(\underset{=}{r}\right)$$

we can rewrite eq 42 as

47 $$\Phi_{R}\left(\underset{=}{r}\right)=\exp\left[\frac{iNm}{M+Nm}K\cdot R\right]\times \exp\left[\frac{ie}{2\hbar c}\sum_{i=1}^{N}\left(B\times r_{i}\right)\cdot R\right]\Pi \left(\underset{=}{r}-R\right)$$

Then, with the definitions of the terms in the Hamiltonian eqs 20 and 21

48 $$\begin{array}{l} \langle \Phi_{R}\left(\underset{=}{r}\right)|{Ĥ}_{e}+{Ĥ}_{ee}+{Ĥ}_{ne}|\Phi_{R}{\left(\underset{=}{r}\right)\rangle }_{\underset{=}{r}}=\\ \sum_{i=1}^{N}\left[-\frac{\hbar^{2}}{2m}\langle \Pi \left(\underset{=}{r}\right)\left|\nabla_{r_{i}}^{2}\right|\Pi {\left(\underset{=}{r}\right)\rangle }_{\underset{=}{r}}-\frac{ie\hbar }{2mc}\langle \Pi \left(\underset{=}{r}\right)\left|\left(B\times r_{i}\right)\cdot \nabla_{r_{i}}\right|\Pi {\left(\underset{=}{r}\right)\rangle }_{\underset{=}{r}}+\frac{e^{2}}{8mc^{2}}\langle \Pi \left(\underset{=}{r}\right)\left|{\left(B\times r_{i}\right)}^{2}\right|\Pi {\left(\underset{=}{r}\right)\rangle }_{\underset{=}{r}}\right]+\\ \langle \Pi \left(\underset{=}{r}\right)|\sum_{i\neq j=1}^{N}\frac{e^{2}}{\left|r_{i}-r_{j}\right|}\left|\Pi {\left(\underset{=}{r}\right)\rangle }_{\underset{=}{r}}-\langle \Pi \left(\underset{=}{r}\right)|\sum_{i=1}^{N}\frac{Ne^{2}}{\left|r_{i}\right|}\right|\Pi {\left(\underset{=}{r}\right)\rangle }_{\underset{=}{r}}=const \end{array}$$

Furthermore, by virtue of eq 9, we have

$$Q\left(R\right)=\frac{\hbar^{2}}{2M}{\left\langle {\left|\frac{ie}{2\hbar c}\sum_{i=1}^{N}\left(B\times r_{i}\right)\phi_{K}\left(\underset{=}{r}-R\right)+\nabla_{R}\phi_{K}\left(\underset{=}{r}-R\right)\right|}^{2}\right\rangle }_{\underset{=}{r}}-\frac{1}{2M}A^{2}\left(R\right)$$

and, after some algebra

49 $$Q\left(R\right)=Q_{0}=const$$

where

50 $$\begin{array}{l} Q_{0}=\frac{e^{2}}{4Mc^{2}}{\left\langle \phi_{K}\left(\underset{=}{r}\right)\left|{\left[\sum_{i=1}^{N}B\times r_{i}\right]}^{2}\right|\phi_{K}\left(\underset{=}{r}\right)\right\rangle }_{\underset{=}{r}}-\frac{e\hbar }{2Mc}Im{\left\langle \phi_{K}\left(\underset{=}{r}\right)\left|\sum_{i,j=1}^{N}\left(B\times r_{i}\right)\nabla_{r_{j}}\right|\phi_{K}\left(\underset{=}{r}\right)\right\rangle }_{\underset{=}{r}}\\ +\frac{\hbar^{2}}{2M}{\left\langle \sum_{i=1}^{N}\nabla_{r_{i}}\phi_{K}\left(\underset{=}{r}\right)|\sum_{i=1}^{N}\nabla_{r_{i}}\phi_{K}\left(\underset{=}{r}\right)\right\rangle }_{\underset{=}{r}}-\frac{1}{2M}A_{0}^{2} \end{array}$$

Collecting eqs 8, 48, and 49, we conclude that

51 $$\epsilon \left(R\right)=\epsilon_{0}=const$$

By showing that both ***A*** ^tot^(***R***) and ϵ^tot^(***R***) are constant in the case of a neutral atom moving in a uniform magnetic field, we have proven the conjecture of Qin et al.^20^ the latter having been based on intuitive arguments. In our proof it was essential that the atom is in one of its eigenstates, while, naturally for a qualitative consideration, ref 20 did not account for the latter restriction. In Section 5 we will show that the conditions for the same result to hold within the BO approximation are much looser than within the exact EF, allowing for an arbitrary nuclear wave-packets, in accord with refs 18 and 19 .

Explicit examples of the violation of the compensation between the two fields are given in Section 6. We also note that the compensation between the physical and Berry curvature magnetic fields depends crucially on the system comprising a bound state. This is (I) understandable from physical arguments, since, obviously, infinitely separated charges experience the nonzero Lorentz force individually and (II) Mathematically this manifests itself in our proof relying on the square integrability of the relative wave function [transition from eqs 43 to 44]. Therefore, the case of noninteracting particles does not constitute a counterexample to our result. On the other hand, let us consider a weak interparticle interaction, but such, that the bound state persists. Our result leads to the important and not an *a priori* obvious conclusion that, for an arbitrarily shallow neutral atom, the compensation property holds. We also note that this rigorous result is not in a logical contradiction with the intuitively plausible picture of the Lorentz force acting on the nucleus locally in time, when the latter is well separated from electrons.

### 3.3 Implications for Equations of Motion

Equations of motion can be simplified in the specific case of this section. Using eqs 26, 41, 46, and 51, we rewrite eqs 24 and 25 as

52 $$i\hbar \partial_{t}\chi \left(R,t\right)=\frac{1}{2M}{\left[-i\hbar \nabla_{R}+A_{0}\right]}^{2}\chi \left(R,t\right)+\epsilon_{0}\chi \left(R,t\right)$$

53 $$i\hbar \partial_{t}\Phi_{R}\left(\underset{=}{r},t\right)=\left({Ĥ}_{BO}-{\mathrm{\epsilon ̃}}_{0}\right)\Phi_{R}\left(\underset{=}{r},t\right)+\frac{1}{2M}{\left[-i\hbar \nabla_{R}-\frac{Ze}{c}A\left(R\right)+{Ã}_{0}\right]}^{2}\Phi_{R}\left(\underset{=}{r},t\right)$$

where

$$\begin{array}{l} {\mathrm{\epsilon ̃}}_{0}=\epsilon_{0}+\frac{1}{2M}{Ã}_{0}^{2}\\ {Ã}_{0}=A_{0}-\frac{i\hbar \nabla_{R}\chi \left(R\right)}{\chi \left(R\right)}=A_{0}+\frac{M}{M+Nm}K \end{array}$$

From the result eq 53 and its derivation we conclude instructively: (I) In the case of an eigenstate of a neutral atom in uniform magnetic field, the electronic equation of motion coincides, to within a gauge transformation, with the original many-body Scrödinger equation; II This property does not hold in the general case.

We further note that, because of the asymmetry of the electronic equation of motion with respect to $A^{tot}$ and A , the residual (***R***-independent) vector potentials $A_{0}$ and ${Ã}_{0}$ in eqs 52 and 53 are different. Due to $A_{0}$ , the nuclear equation of motion eq 52 does not possess the time-reversal symmetry despite the Berry curvature and the physical magnetic fields compensation. However, since $A_{0}$ is constant, this time-reversal symmetry breaking is apparent, and it can be gauged out by a suitable transformation eq 7. In contrast, the lack of the time-reversal symmetry in the electronic equation of motion eq 53 is essential due to the ${Ĥ}_{e}$ part of the Hamiltonian, which contains the physical magnetic field.

We, finally, note that it might seem tempting to nullify the force on the nucleus without the evaluation of the Berry connection vector potential, by noting that, if the force were nonzero, then the momentum would be changing, which cannot be happening in an eigenstate. There is, however, a danger in using such kind of argumentation in the case of an eigenstate of the continuous spectrum, as is our case here. We illustrate this with a simple example of a two body problem in the field-free case. The eigenstate wave function can be written as

$$\Psi \left(R_{c},\mathrm{r̃}\right)=e^{i/\hbar P\cdot R_{c}}\phi \left(\mathrm{r̃}\right)$$

where ***R***_*c*_ and ***r̃*** are the c.m. and relative coordinates, respectively. According to eq 4, the force on the particle 1 is

54 $$\begin{array}{l} f_{1}=-\int |\Psi (\left(Mr_{1}+mr_{2}\right)/\left(M+m\right),r_{1}-r_{2}{|}^{2}\\ \times \nabla_{r_{1}}v\left(|r_{1}-r_{2}|\right)dr_{1}dr_{2}=-\int |\Psi (R_{c},\mathrm{r̃}{|}^{2}\nabla_{\mathrm{r̃}}v\left(\mathrm{r̃}\right)dR_{c}d\mathrm{r̃}\\ =-\int 1dR_{c}\int |\phi \left(\mathrm{r̃}\right){|}^{2}\nabla_{\mathrm{r̃}}v\left(\mathrm{r̃}\right)d\mathrm{r̃} \end{array}$$

where v(r̃) is the interparticle interaction potential. The first integral in the third line of eq 54 is infinity, while the second one is zero, since ϕ(r̃) can be chosen even or odd. The force, therefore, is undetermined, which demonstrates the difficulty in the application of eq 4 in the case of an eigenstate of the continuous spectrum.

On the other hand, our proof of the mutual cancellation of the Berry curvature and physical uniform magnetic field in the case of the continuous spectrum eigenstate does not suffer from the above ambiguity, and it should be given a preference.

## Residual Berry Connection Vector Potential $A_{0}$

4

We have demonstrated the exact compensation between the physical magnetic and the Berry curvature fields in the case of an eigenstate of a moving neutral atom. We have also observed the remaining uncompensated constant (residual) Berry connection vector potential $A_{0}$ , the latter, obviously, irrelevant to the Berry curvature field. Nonetheless, $A_{0}$ is of consequence, since it affects the gauge-invariant current density of eq 27. In the case under consideration

$$J=\frac{\hbar }{M}Im\chi \left(R\right)\nabla_{R}\chi \left(R\right)+\frac{1}{M}|\chi \left(R\right){|}^{2}A_{0}=\frac{1}{M+Nm}K+\frac{1}{M}A_{0}$$

where $A_{0}$ is given by eq 45.

In the following subsection, we obtain and analyze an explicit expression for $A_{0}$ in the case of a model Harmonium atom in magnetic field.

### 4.1 Harmonium Atom in Magnetic Field

The eigenvalue problem for the separated relative Hamiltonian of the hydrogen-like atom in uniform magnetic field reads^23^

$$\begin{array}{l} {Ĥ}_{rel}\phi_{K}\left(r\right)=[\frac{K^{2}}{2M_{c}}+\frac{e}{cM_{c}}\left(K\times B\right)\cdot r+\frac{1}{2\mu }{\mathrm{p̂}}^{2}\\ +\frac{e}{2c}\left(\frac{1}{m}-\frac{1}{M}\right)B\cdot \left(r\times \mathrm{p̂}\right)+\frac{e^{2}}{8\mu c^{2}}{\left(B\times r\right)}^{2}+V\left(r\right)]\phi_{K}\left(r\right)=E_{K}\phi_{K}\left(r\right) \end{array}$$

where μ = *Mm*/*M*_*c*_ is the reduced mass. In the case of the harmonic interparticle potential

$$V\left(r\right)=\frac{1}{2}\mu \omega_{0}^{2}r^{2}$$

this problem admits the explicit solution^23^

55 $$\phi_{K}\left(r\right)=\exp\left(i\frac{M-m}{2\hbar M_{c}}\alpha K_{⊥}\cdot r\right)\phi_{0K}\left(r-\alpha r_{0}\right)$$

where

$$\begin{array}{l} r_{0}=-cK\times B/\left(eB^{2}\right)\\ \alpha ={\left(1+\frac{Mmc^{2}\omega_{0}^{2}}{e^{2}B^{2}}\right)}^{-1} \end{array}$$

and ϕ_0**K**_(***r*** ′) is the wave function of the harmonic oscillator in magnetic field problem

56 $$\begin{array}{l} \{\frac{K_{z}^{2}}{2M_{c}}+\frac{1}{2}\mu \omega_{0}^{2}z^{2}-\frac{\hbar^{2}}{2\mu }\frac{\partial^{2}}{\partial z^{2}}-\frac{\hbar^{2}}{2\mu }\left(\frac{\partial^{2}}{\partial {x^{\prime }}^{2}}+\frac{\partial^{2}}{\partial {y^{\prime }}^{2}}\right)-\frac{ie\hbar }{2c}\left(\frac{1}{m}-\frac{1}{M}\right)B\left(x^{\prime }\frac{\partial }{\partial y^{\prime }}-y^{\prime }\frac{\partial }{\partial x^{\prime }}\right)\\ +\left(\frac{e^{2}}{8\mu c^{2}}B^{2}+\frac{1}{2}\mu \omega_{0}^{2}\right)\left({x^{\prime }}^{2}+{y^{\prime }}^{2}\right)+\frac{K_{⊥}^{2}}{2M_{c}}\left(1-\alpha \right)\}\phi_{0K}\left(r^{\prime }\right)=E_{K}\phi_{0K}\left(r^{\prime }\right) \end{array}$$

Although the analytical eigenfunctions of the problem eq 56 are known, here we do not need them explicitly. It is enough to note that the Hamiltonian in eq 56 commutes with the inversion operator and, therefore, the eigenfunctions can be classified into the even and odd ones. By the substitution of eqs 55 into [Disp-formula eq45] we immediately have

57 $$A_{0}=-\alpha \frac{M}{M+m}K_{⊥}$$

where ϕ_0**K**_(***r*** ′) has disappeared due to the parity and the normalization. For the nuclear current density we have by virtue of eq 27

$$J=\frac{1}{M+m}\left[K_{∥}+\left(1-\alpha \right)K_{⊥}\right]$$

where we have normalized the nuclear particle density to unity. Obviously, the vectors of the current-density and the pseudomomentum are not parallel to each other in the general case.

An eigenstate of an atom in uniform magnetic field carries nuclear (and electronic) current, with the direction of the latter noncollinear to either the magnetic field or to the pseudomomentum. In the example of this section, the degree of the noncollinearity can be conveniently characterized by the quantity $A_{0}/K_{⊥}$ . Figures 1 and 2 demonstrate the sensitivity of this quantity to the ratio of masses of the two particles and to the strength of magnetic field, respectively.

**Figure 1:**
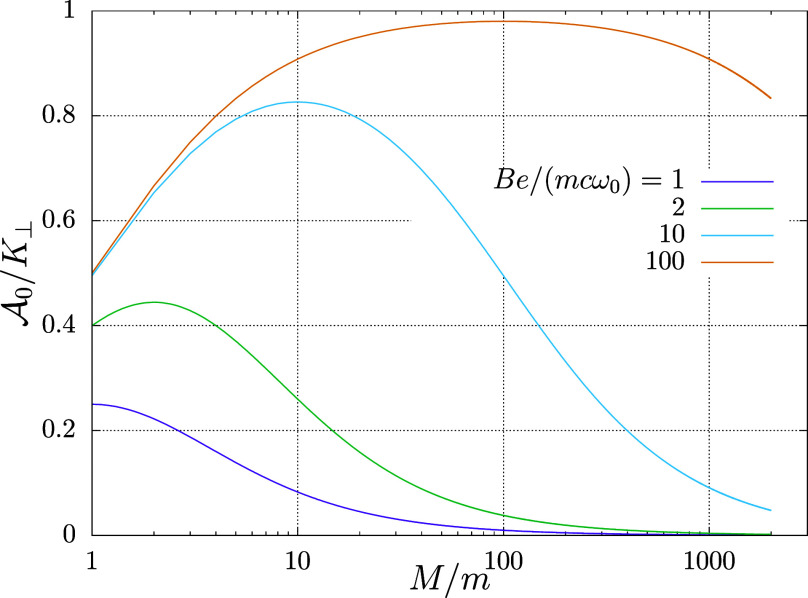
Harmonium atom in magnetic field. Coefficient of proportionality between the residual Berry connection vector potential $A_{0}$ and the normal-to-the-magnetic-field component of the pseudomomentum **K**_⊥_ [eq 57] versus the ratio of masses *M*/*m* of the two particles. Results for four magnitudes of the magnetic field, in the units of *mc*ω_0_/*e*, are shown.

**Figure 2:**
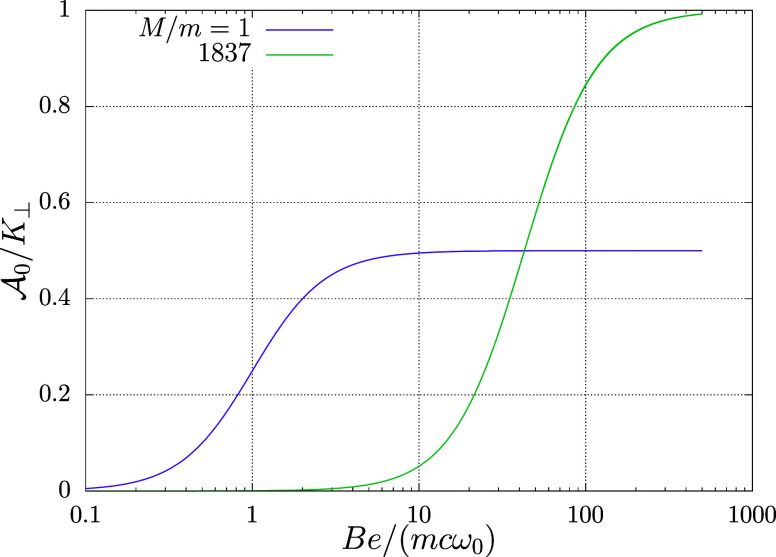
Harmonium atom in magnetic field. Coefficient of proportionality between the residual Berry connection vector potential $A_{0}$ and the normal-to-the-magnetic-field component of the pseudomomentum **K**_⊥_ [eq 57] versus the magnitude of the magnetic field, the latter in the units of *mc*ω_0_/*e*. Results for two values of the ratio of the masses of the particles are shown.

## 5 Born–Oppenheimer Approximation

The purpose of this section is to show that, within the BO approximation, for the compensation between the Berry curvature and physical magnetic fields the requirement of the atom being in an eigenstate is lifted. It is rather sufficient that the atom moves on a single BO surface, while χ­(***R***, *t*) can be an arbitrary wave packet propagating in accordance with its equation of motion (eq 24). This is due to the equation for $\Phi_{R}\left(\underset{=}{r}\right)$ not depending on χ­(***R***, *t*) in the BO case, so that the potentials in the equation for χ do not depend on χ itself. The latter is not the case in the nonadiabatic dynamics, where the conditions for the compensation are more restrictive.

Within the BO approximation

58 $$\Psi^{BO}\left(R,\underset{=}{r},t\right)=\chi^{BO}\left(R,t\right)\Phi_{R}^{BO}\left(\underset{=}{r}\right)$$

where $\Phi_{R}^{BO}\left(\underset{=}{r}\right)$ is an eigenfunction of the equation

59 $$\begin{array}{l} \{\sum_{i=1}^{N}\left[-\frac{\hbar^{2}}{2m}\nabla_{r_{i}}^{2}-\frac{i\hbar e}{2mc}\left(B\times r_{i}\right)\cdot \nabla_{r_{i}}+\frac{e^{2}}{8mc^{2}}{\left(B\times r_{i}\right)}^{2}\right]\\ +\sum_{i\neq j=1}^{N}\frac{e^{2}}{\left|r_{i}-r_{j}\right|}-\sum_{i=1}^{N}\frac{Ne^{2}}{\left|r_{i}-R\right|}\}\Phi_{R}^{BO}\left(\underset{=}{r}\right)=E_{R}\Phi_{R}^{BO}\left(\underset{=}{r}\right) \end{array}$$

By the substitution

60 $$\Phi_{R}^{BO}\left(\underset{=}{r}\right)=\Pi_{R}^{BO}\left(\underset{=}{r}\right)\exp\left[-\frac{ie}{2\hbar c}\sum_{i=1}^{N}\left(B\times R\right)\cdot r_{i}\right]$$

we rewrite eq 59 as

61 $$\begin{array}{l} \left\{\sum_{i=1}^{N}[-\frac{\hbar^{2}}{2m}\nabla_{r_{i}}^{2}-\frac{i\hbar e}{2mc}\left[B\times \left(r_{i}-R\right)\right]\cdot \nabla_{r_{i}}+\frac{e^{2}}{8mc^{2}}\times [({B\times \left(r_{i}-R\right)]}^{2}\right]\\ +\sum_{i\neq j=1}^{N}\frac{e^{2}}{\left|r_{i}-r_{j}\right|}-\sum_{i=1}^{N}\frac{Ne^{2}}{\left|r_{i}-R\right|}\}\Pi_{R}^{BO}\left(\underset{=}{r}\right)=E_{R}\Pi_{R}^{BO}\left(\underset{=}{r}\right) \end{array}$$

From eq 61 we conclude that $\Pi_{R}\left(\underset{=}{r}\right)$ is a function of $\underset{=}{r}-R$ only, and then

$$\Phi_{R}^{BO}\left(\underset{=}{r}\right)=\Pi^{BO}\left(\underset{=}{r}-R\right)\exp\left[-\frac{ie}{2\hbar c}\sum_{i=1}^{N}\left(B\times R\right)\cdot r_{i}\right]$$

which can also be written as

62 $$\Phi_{R}^{BO}\left(\underset{=}{r}\right)=\Pi^{BO}\left(\underset{=}{r}-R\right)\exp\left[\frac{ie}{2\hbar c}\sum_{i=1}^{N}\left(B\times r_{i}\right)\cdot R\right]$$

With the use of eq 62, the whole sequence of the derivations of Sections 3.1 and 3.2 can be repeated literally, leading to the result that in the BO approximation, as well as in EF, the nucleus of a neutral atom moves in a uniform magnetic field as a free particle, however, in contrast with the exact EF result, its wave packet χ^BO^(***R***, *t*) in eq 58 can be arbitrary, and only the confinement to a single BO surface is a requirement.

## 6 Counterexamples of Wave-Packets Violating the Berry Curvature Compensation Condition

In this section we demonstrate two cases of the breaking of the mutual compensation between the Berry curvature and physical magnetic fields. Our intention in the first case is to show that the compensation concluded on the basis of the BO approximation may be spurious, being an artifact of that approximation. In the second case we construct a wave packet which violates the compensation within both the exact theory and the BO approximation.

To maximally simplify the construction of counterexamples, it is legitimate to consider the field-free cases. Indeed, arriving at finite Berry curvature fields, we conclude that they are not compensated by the zero physical magnetic field, which is sufficient for the purpose of our proofs.

### 6.1 Case I: Compensation within BO Approximation Holds but It Is Broken by the Nonadiabaticity of the Exact Dynamics

In the field-free case, we consider a hydrogenic atom at the relative motion eigenstate ϕ­(***r***–***R***), which we assume real, and the eigenenergy ϵ_0_, and we consider an arbitrary the c.m. wave packet *f*(**P**_*c*_), resulting in the exact two-body wave function

$$\Psi \left(r,R,t\right)=\phi \left(r-R\right)e^{-i\epsilon_{0}t/\hbar }\int f\left(P_{c}\right)e^{iP_{c}\cdot (MR+mr)/\hbar M_{c}}e^{-iP_{c}^{2}t/2\hbar M_{c}}dP_{c}$$

Within EF, we split Ψ­(***r***, ***R***, *t*) in the following way

$$\begin{array}{l} \chi \left(R,t\right)={\left[\int |\Psi \left(r,R,t\right){|}^{2}dr\right]}^{1/2}\\ \Phi_{R}\left(r,t\right)=\frac{\Psi \left(r,R,t\right)}{\chi \left(R,t\right)} \end{array}$$

Then

63 $$\chi \left(R,t\right)={\left\{\int W\left(P_{c},P_{c}^{\prime };R,t\right)dP_{c}dP_{c}^{\prime }\right\}}^{1/2}$$

where

64 $$W\left(P_{c},P_{c}^{\prime };R,t\right)=f\left(P_{c}\right)f^{*}\left(P_{c}^{\prime }\right)e^{i/\hbar \left(P_{c}-P_{c}^{\prime }\right)\cdot R}G\left[\frac{m}{\hbar M_{c}}\left(P_{c}-P_{c}^{\prime }\right)\right]e^{i(P_{c}^{\prime 2}-P_{c}^{2})/2\hbar M_{c}t}$$

65 $$G\left(p\right)=\int e^{ip\cdot \mathrm{r̃}}\phi^{2}\left(\mathrm{r̃}\right)d\mathrm{r̃}$$

According to eqs 10 and 6

66 $$A\left(R,t\right)=-i\hbar \frac{{\left\langle \Psi \left|\nabla_{R}\right|\Psi \right\rangle }_{r}}{\chi^{2}\left(R,t\right)}+i\hbar \frac{\nabla_{R}\chi \left(R,t\right)}{\chi \left(R,t\right)}$$

In Appendix B, by working out expression eq 66, we obtain for the Berry curvature

67 $$\begin{array}{l} B\left(R,t\right)=\nabla_{R}\times A\left(R,t\right)=\frac{M}{\hbar M_{c}}Im[\frac{1}{\chi^{4}\left(R,t\right)}\int P_{c}W\left(P_{c},P_{c}^{\prime };R,t\right)dP_{c}dP_{c}^{\prime }\times \int P_{c}^{\prime }W\left(P_{c},P_{c}^{\prime };R,t\right)dP_{c}dP_{c}^{\prime }\\ -\frac{1}{\chi^{2}\left(R,t\right)}\int P_{c}\times P_{c}^{\prime }W\left(P_{c},P_{c}^{\prime };R,t\right)dP_{c}dP_{c}^{\prime }] \end{array}$$

For the BO approximation, in eq 67 we take the limit *M* → ∞ and note that, according to the definition eq 65, *G*(**0**) = 1. Then, after some algebra, eq 67 reduces to zero identically, which proves that there is no net Berry curvature in our example within BO approximation. To show that this is not, generally, the case within EF, for the wave packet we consider a superposition of three plane-waves (interestingly, two are not enough to construct a finite B )­

68 $$f\left(P\right)=C_{1}\delta \left(P-P_{1}\right)+C_{2}\delta \left(P-P_{2}\right)+C_{3}\delta \left(P-P_{3}\right)$$

After extensive algebra, eqs 67 and 63 then evaluate to

69 $$\begin{array}{l} B\left(R,t\right)=\frac{2MC_{1}C_{2}C_{3}}{\hbar M_{c}\chi^{4}\left(R,t\right)}\left(P_{1}\times P_{2}+P_{3}\times P_{1}+P_{2}\times P_{3}\right)\times \{\\ \left[G\left[\frac{m}{\hbar M_{c}}\left(P_{2}-P_{1}\right)\right]-G\left[\frac{m}{\hbar M_{c}}\left(P_{3}-P_{1}\right)\right]G\left[\frac{m}{\hbar M_{c}}\left(P_{3}-P_{2}\right)\right]\right]C_{3}\;\sin\left[\frac{P_{2}-P_{1}}{\hbar }\cdot R-\frac{P_{2}^{2}-P_{1}^{2}}{2\hbar M_{c}}t\right]\\ -\left[G\left[\frac{m}{\hbar M_{c}}\left(P_{3}-P_{1}\right)\right]-G\left[\frac{m}{\hbar M_{c}}\left(P_{2}-P_{1}\right)\right]G\left[\frac{m}{\hbar M_{c}}\left(P_{3}-P_{2}\right)\right]\right]C_{2}\;\sin\left[\frac{P_{3}-P_{1}}{\hbar }\cdot R-\frac{P_{3}^{2}-P_{1}^{2}}{2\hbar M_{c}}t\right]\\ +\left[G\left[\frac{m}{\hbar M_{c}}\left(P_{3}-P_{2}\right)\right]-G\left[\frac{m}{\hbar M_{c}}\left(P_{2}-P_{1}\right)\right]G\left[\frac{m}{\hbar M_{c}}\left(P_{3}-P_{1}\right)\right]\right]C_{1}\;\sin\left[\frac{P_{3}-P_{2}}{\hbar }\cdot R-\frac{P_{3}^{2}-P_{2}^{2}}{2\hbar M_{c}}t\right]\} \end{array}$$

$$\begin{array}{l} \chi^{2}\left(R,t\right)=C_{1}^{2}+C_{2}^{2}+C_{3}^{2}+2C_{1}C_{2}G\left[\frac{m}{\hbar M_{c}}\left(P_{2}-P_{1}\right)\right]\cos\left[\frac{P_{2}-P_{1}}{\hbar }\cdot R-\frac{P_{2}^{2}-P_{1}^{2}}{2\hbar M_{c}}t\right]\\ +2C_{1}C_{3}G\left[\frac{m}{\hbar M_{c}}\left(P_{3}-P_{1}\right)\right]\cos\left[\frac{P_{3}-P_{1}}{\hbar }\cdot R-\frac{P_{3}^{2}-P_{1}^{2}}{2\hbar M_{c}}t\right]\\ +2C_{2}C_{3}G\left[\frac{m}{\hbar M_{c}}\left(P_{3}-P_{2}\right)\right]\cos\left[\frac{P_{3}-P_{2}}{\hbar }\cdot R-\frac{P_{3}^{2}-P_{2}^{2}}{2\hbar M_{c}}t\right] \end{array}$$

It can be seen from eq 69 that the Berry curvature B(R,t) of our example is, generally speaking, nonzero and time-dependent. Q.E.D.

### 6.2 Case II: Violation of the Compensation within Both BO Approximation and the Exact Nonadiabatic Theory

Here we demonstrate that, for general wave-packets, the cancellation between the physical magnetic field and the Berry curvature does not take place. A simple and clear counterexample to the cancellation is to demonstrate a wave packet which, in the absence of the magnetic field, supports a finite Berry curvature. To do this, we consider hydrogen atom and construct a superposition of two eigenstates

$$\Psi \left(R,r,t\right)=\frac{1}{\sqrt{2}}\sum_{j=1}^{2}e^{i/\hbar P_{j}\cdot (MR+mr)/M+m}e^{-iE_{j}/\hbar t}\phi_{j}\left(r-R\right)$$

where *E*_*j*_ and **P**_*j*_ are the energies and c.m. momenta, respectively, of the corresponding states, and ϕ_*j*_(***r*** – ***R***) are two eigenstates of the relative motion of the particles, which we choose real. Then

70 $${\left\langle \Psi \left(R,r,t\right)|\Psi \left(R,r,t\right)\right\rangle }_{r}=1+Ree^{i/\hbar \left(E_{2}-E_{1}\right)t}e^{i/\hbar \left(P_{1}-P_{2}\right)\cdot R}f\left[\frac{m\left(P_{1}-P_{2}\right)}{\hbar \left(M+m\right)}\right]$$

where

$$f\left(q\right)=\int e^{iq\cdot r}\phi_{1}\left(r\right)\phi_{2}\left(r\right)\;dr$$

Along the lines of EF, we construct Φ_***R***_(***r***, *t*) as

$$\Phi_{R}\left(r,t\right)=\Psi \left(R,r,t\right){\left\langle \Psi \left(R,r,t\right)|\Psi \left(R,r,t\right)\right\rangle }_{r}^{-1/2}$$

which clearly satisfies the partial normalization requirement (eq 6). Then, after some algebra, the Berry connection evaluates to

$$\begin{array}{l} A\left(R,t\right)=-i\hbar \langle \Phi_{R}\left(r,t\right)|\nabla_{R}|\Phi_{R}\left(r,t\right)\rangle =\frac{M}{2M_{c}}\left(P_{1}+P_{2}\right)\\ +\frac{\hbar }{2}\langle \Psi \left(R,r,t\right)|\Psi {\left(R,r,t\right)\rangle }_{r}^{-1}Ime^{i/\hbar \left(E_{2}-E_{1}\right)t}e^{i/\hbar \left(P_{1}-P_{2}\right)\cdot R}G\left[\frac{m\left(P_{1}-P_{2}\right)}{\hbar M_{c}}\right] \end{array}$$

where

$$G\left(q\right)=\int e^{iq\cdot r}\left[\phi_{1}\left(r\right)\nabla_{r}\phi_{2}\left(r\right)-\phi_{2}\left(r\right)\nabla_{r}\phi_{1}\left(r\right)\right]\;dr$$

Accordingly, for the Berry curvature we can write

$$\begin{array}{l} B\left(R,t\right)=\nabla_{R}\times A\left(R,t\right)=\frac{1}{2}{\left\langle \Psi \left(R,r,t\right)|\Psi \left(R,r,t\right)\right\rangle }_{r}^{-2}\left(P_{1}-P_{2}\right)\times Re\{[e^{i/\hbar \left(E_{2}-E_{1}\right)t}e^{i/\hbar \left(P_{1}-P_{2}\right)\cdot R}\\ +f^{*}\left[\frac{m\left(P_{1}-P_{2}\right)}{\hbar \left(M+m\right)}\right]]G\left[\frac{m\left(P_{1}-P_{2}\right)}{\hbar \left(M+m\right)}\right]\} \end{array}$$

Let ϕ_1_ and ϕ_2_ be the first s- and p-states of the atom, respectively. Then^21^

$$\begin{array}{l} \phi_{1}\left(r\right)=\frac{1}{\sqrt{2\pi }}e^{-\mathrm{r̃}}\\ \phi_{2}\left(r\right)=\frac{1}{4\sqrt{2\pi }}\mathrm{r̃}e^{-\mathrm{r̃}/2}\;\cos\theta \end{array}$$

where $\mathrm{r̃}=\frac{\mu e^{2}}{\hbar^{2}}r$ and μ is the reduced mass. Functions *f*(**q**) and **G**(**q**) are straightforwardly evaluated to

71 $$\begin{array}{l} f\left(q\right)=\frac{384i{\mathrm{q̃}}_{z}}{{\left(9+4{\mathrm{q̃}}^{2}\right)}^{3}}\\ G\left(q\right)=\frac{i}{3}f\left(q\right)q+\frac{\mu e^{2}}{\hbar^{2}}\frac{32}{{\left(9+4{\mathrm{q̃}}^{2}\right)}^{2}}ẑ \end{array}$$

where $\mathrm{q̃}=\frac{\hbar^{2}}{\mu e^{2}}q$ and **ẑ** is the unit vector along the *z*-axis. Then

$$\begin{array}{l} B\left(R,t\right)=\frac{16\mu e^{2}}{\hbar^{2}}{\left\langle \Psi \left(R,r,t\right)|\Psi \left(R,r,t\right)\right\rangle }_{r}^{-2}\left[\left(P_{1}-P_{2}\right)\times ẑ\right]\\ \times \cos\left\{\frac{1}{\hbar }\left[\left(E_{2}-E_{1}\right)t+\left(P_{1}-P_{2}\right)\cdot R\right]\right\}\frac{1}{{\left(9+4{\mathrm{q̃}}^{2}\right)}^{2}} \end{array}$$

Let, for further simplification, **P**_1_–**P**_2_ be aligned along the *x*-axis. Then, according to eqs 70 and 71, the last equation reduces to

72 $$H_{y}\left(R,t\right)=-\frac{16\mu e^{2}}{\hbar^{2}}\frac{\left(P_{1x}-P_{2x}\right)}{9+\frac{4\hbar^{2}}{M^{2}e^{4}}{\left(P_{1x}-P_{2x}\right)}^{2}}\cos\left\{\frac{1}{\hbar }\left[\left(E_{2}-E_{1}\right)t+\left(P_{1x}-P_{2x}\right)R_{x}\right]\right\}$$

Equation 72 concludes the construction of an example of the Berry curvature, which being finite, is not compensated by the physical magnetic field, the latter being zero in this example. Nor $B_{y}\left(R,t\right)$ is zero in the BO approximation, as can be seen by taking the *M* → ∞ limit in eq 72. To demonstrate the latter facts has been the purpose of this section.

## 7 Conclusions

We have included an external electromagnetic field in the framework of the Exact Factorization formalism. The resulting equations of motion, those for nuclei and electrons, contain both the physical and the Berry connection vector potentials. We have shown, that nuclei move under the action of the sum of the two vector potentials, while for electrons, the two potentials enter the equation of motion in a more complicated, asymmetric way.

As an important particular case, we have applied the EF formalism to an eigenstate of a neutral atom with the moving center of mass. As an exact result, we have proven that the nucleus of the atom moves as a free particle. The latter arises as a result of the exact compensation, in the nuclear equation of motion, between the physical and the Berry connection vector potentials. We have illustrated the general theory with the analytical solution for the Harmonium atom in magnetic field, where the quantities relevant to EF formalism admit explicit evaluation and visualization. At the same time, we have demonstrated an important distinction from the corresponding result within the Born–Oppenheimer approximation, where the compensation between the two fields holds at the less restrictive condition of an arbitrary nuclear wave packet on a single BO surface.

Our results being based on the exact theory rather than relying on specific approximations, they provide insights in the Exact Factorization method as a whole.

## Appendix A

### A Derivation of Equations of Motion

#### A.1 Nuclear Equation of Motion Equation 24

Provisionally considering $\Phi_{\underset{=}{R}}\left(\underset{=}{r},t\right)$ as fixed, we apply the McLachlan’s time-dependent variational principle^4^ to determine $\chi \left(\underset{=}{R},t\right)$ . Although the use of the McLachlan’s variational principle and the Frenkel’s action principle^24^ are equivalent in the context of this work, and although the latter has mostly been employed in the EF literature before, we give preference to the former one, since it is a minimal rather than stationary variational principle, free of the shortcomings inherent to the latter one.^4^ The essence of the variational principle of McLachlan is that, at every time moment *t*, one considers the wave function fixed, while its time-derivative, which determines its value at the infinitesimal close time moment *t* + δ*t*, is tuned to minimize, in the *L*_2_ norm, the difference between the LHS and RHS of the Schrödinger equation.

At every time moment *t*, we minimize the functional

$$\Delta \left(t\right)=\int {\left|\left[i\hbar \partial_{t}-Ĥ\left(t\right)\right]\chi \left(\underset{=}{R},t\right)\Phi_{\underset{=}{R}}\left(\underset{=}{r},t\right)\right|}^{2}d\underset{=}{R}d\underset{=}{r}$$

with respect to the variation of $\partial_{t}\chi \left(\underset{=}{R},t\right)$ . Equating the variation of the functional to zero, we write

$$\begin{array}{l} \int \Phi_{\underset{=}{R}}^{*}\left(\underset{=}{r},t\right)\{i\hbar \Phi_{\underset{=}{R}}\left(\underset{=}{r},t\right)\partial_{t}\chi \left(\underset{=}{R},t\right)+i\hbar \chi \left(\underset{=}{R},t\right)\partial_{t}\Phi_{\underset{=}{R}}\left(\underset{=}{r},t\right)\\ -Ĥ\left(t\right)\left[\chi \left(\underset{=}{R},t\right)\Phi_{\underset{=}{R}}\left(\underset{=}{r},t\right)\right]\}\delta \partial_{t}\chi^{*}\left(\underset{=}{R},t\right)\;d\underset{=}{R}d\underset{=}{r}=0 \end{array}$$

Considering the arbitrariness of $\delta \partial_{t}\chi^{*}\left(\underset{=}{R},t\right)$ , we can further write

$$\int \Phi_{\underset{=}{R}}^{*}\left(\underset{=}{r},t\right)\left\{i\hbar \Phi_{\underset{=}{R}}\left(\underset{=}{r},t\right)\partial_{t}\chi \left(\underset{=}{R},t\right)+i\hbar \chi \left(\underset{=}{R},t\right)\partial_{t}\Phi_{\underset{=}{R}}\left(\underset{=}{r},t\right)-Ĥ\left(t\right)\left[\chi \left(\underset{=}{R},t\right)\Phi_{\underset{=}{R}}\left(\underset{=}{r},t\right)\right]\right\}\;d\underset{=}{r}=0$$

or, with account of the normalization condition (6)

$$i\hbar \partial_{t}\chi \left(\underset{=}{R},t\right)=\int \Phi_{\underset{=}{R}}^{*}\left(\underset{=}{r},t\right)\left\{Ĥ\left(t\right)\left[\chi \left(\underset{=}{R},t\right)\Phi_{\underset{=}{R}}\left(\underset{=}{r},t\right)\right]-i\hbar \chi \left(\underset{=}{R},t\right)\partial_{t}\Phi_{\underset{=}{R}}\left(\underset{=}{r},t\right)\right\}\;d\underset{=}{r}$$

which can be rewritten as

73
$$\begin{array}{l} i\hbar \partial_{t}\chi \left(\underset{=}{R},t\right)={\left\langle \Phi_{\underset{=}{R}}\left(\underset{=}{r},t\right)\left|{Ĥ}_{n}\left(t\right)\right|\chi \left(\underset{=}{R},t\right)\Phi_{\underset{=}{R}}\left(\underset{=}{r},t\right)\right\rangle }_{\underset{=}{r}}\\ +\langle \Phi_{\underset{=}{R}}\left(\underset{=}{r},t\right)|{Ĥ}_{BO}\left(t\right)-i\hbar \partial_{t}|\Phi_{\underset{=}{R}}{\left(\underset{=}{r},t\right)\rangle }_{\underset{=}{r}}\chi \left(\underset{=}{R},t\right) \end{array}$$

where ${Ĥ}^{BO}\left(t\right)$ is defined by eq 21.

Applying explicitly expression (eq 22) of the operator ${Ĥ}_{n}\left(t\right)$ on RHS of eq 73, after a straightforward algebra, we arrive at the nuclear equation of motion (eq 24).

#### A.2 Electronic Equation of Motion Equation 25

Combining eqs 1, 5, and 20, we can write

74
$$i\hbar \partial_{t}\Phi_{\underset{=}{R}}\left(\underset{=}{r},t\right)=\left[{Ĥ}_{BO}\left(t\right)-i\hbar \frac{\partial_{t}\chi \left(\underset{=}{R},t\right)}{\chi \left(\underset{=}{R},t\right)}\right]\Phi_{\underset{=}{R}}\left(\underset{=}{r},t\right)+\frac{1}{\chi \left(\underset{=}{R},t\right)}{Ĥ}_{n}\left(t\right)\left[\chi \left(\underset{=}{R},t\right)\Phi_{\underset{=}{R}}\left(\underset{=}{r},t\right)\right]$$

Equation 25 is, finally, established by the substitution of $\partial_{t}\chi \left(\underset{=}{R},t\right)$ from eq 24 and working out the direct application of ${Ĥ}_{n}\left(t\right)$ of eq 22 on RHS of eq 74.

## Appendix B

### B Derivation of Equation 67

By eq 66 we can write

$$\begin{array}{l} A\left(R,t\right)=\frac{i\hbar }{2\chi^{2}\left(R,t\right)}\int \nabla_{r}\phi^{2}\left(r-R\right)f\left(P_{c}\right)f^{*}\left(P_{c}^{\prime }\right)e^{i\left(P_{c}-P_{c}^{\prime }\right)\cdot (MR+mr)/\hbar M_{c}}e^{i\left(P_{c}^{\prime 2}-P_{c}^{2}\right)t/2\hbar M_{c}}dP_{c}dP_{c}^{\prime }dr\\ +\frac{1}{\chi^{2}\left(R,t\right)}\frac{M}{M_{c}}\int \phi^{2}\left(r-R\right)P_{c}f\left(P_{c}\right)f^{*}\left(P_{c}^{\prime }\right)e^{i\left(P_{c}-P_{c}^{\prime }\right)\cdot (MR+mr)/\hbar M_{c}}e^{i\left(P_{c}^{\prime 2}-P_{c}^{2}\right)t/2\hbar M_{c}}dP_{c}dP_{c}^{\prime }dr+i\hbar \frac{\nabla_{R}\chi \left(R,t\right)}{\chi \left(R,t\right)} \end{array}$$

Or, integrating by parts in ***r*** in the first integral

$$\begin{array}{l} A\left(R,t\right)=\frac{1}{2\chi^{2}\left(R,t\right)}\frac{m}{M_{c}}\int \phi^{2}\left(r-R\right)\left(P_{c}-P_{c}^{\prime }\right)f\left(P_{c}\right)f^{*}\left(P_{c}^{\prime }\right)e^{i\left(P_{c}-P_{c}^{\prime }\right)\cdot (MR+mr)/\hbar M_{c}}e^{i\left(P_{c}^{\prime 2}-P_{c}^{2}\right)t/2\hbar M_{c}}dP_{c}dP_{c}^{\prime }dr\\ +\frac{1}{\chi^{2}\left(R,t\right)}\frac{M}{M_{c}}\int \phi^{2}\left(r-R\right)P_{c}f\left(P_{c}\right)f^{*}\left(P_{c}^{\prime }\right)e^{i\left(P_{c}-P_{c}^{\prime }\right)\cdot (MR+mr)/\hbar M_{c}}e^{i\left(P_{c}^{\prime 2}-P_{c}^{2}\right)t/2\hbar M_{c}}dP_{c}dP_{c}^{\prime }dr+i\hbar \frac{\nabla_{R}\chi \left(R,t\right)}{\chi \left(R,t\right)} \end{array}$$

Then, with the use of eqs 63–65

$$\begin{array}{l} A\left(R,t\right)=\frac{1}{2\chi^{2}\left(R,t\right)}\frac{m}{M_{c}}\int G\left[\frac{m}{\hbar M_{c}}\left(P_{c}-P_{c}^{\prime }\right)\right]\left(P_{c}-P_{c}^{\prime }\right)f\left(P_{c}\right)f^{*}\left(P_{c}^{\prime }\right)e^{i/\hbar \left(P_{c}-P_{c}^{\prime }\right)\cdot R}e^{i\left(P_{c}^{\prime 2}-P_{c}^{2}\right)t/2\hbar M_{c}}dP_{c}dP_{c}^{\prime }\\ +\frac{1}{\chi^{2}\left(R,t\right)}\frac{M}{M_{c}}\int G\left[\frac{m}{\hbar M_{c}}\left(P_{c}-P_{c}^{\prime }\right)\right]P_{c}f\left(P_{c}\right)f^{*}\left(P_{c}^{\prime }\right)e^{i/\hbar \left(P_{c}-P_{c}^{\prime }\right)\cdot R}e^{i\left(P_{c}^{\prime 2}-P_{c}^{2}\right)t/2\hbar M_{c}}dP_{c}dP_{c}^{\prime }+i\hbar \frac{\nabla_{R}\chi \left(R,t\right)}{\chi \left(R,t\right)}\\ =\frac{1}{2\chi^{2}\left(R,t\right)}\frac{m}{M_{c}}\int G\left[\frac{m}{\hbar M_{c}}\left(P_{c}-P_{c}^{\prime }\right)\right]\left(P_{c}-P_{c}^{\prime }\right)f\left(P_{c}\right)f^{*}\left(P_{c}^{\prime }\right)e^{i/\hbar \left(P_{c}-P_{c}^{\prime }\right)\cdot R}e^{i\left(P_{c}^{\prime 2}-P_{c}^{2}\right)t/2\hbar M_{c}}dP_{c}dP_{c}^{\prime }\\ +\frac{1}{\chi^{2}\left(R,t\right)}\frac{M}{M_{c}}\int G\left[\frac{m}{\hbar M_{c}}\left(P_{c}-P_{c}^{\prime }\right)\right]P_{c}f\left(P_{c}\right)f^{*}\left(P_{c}^{\prime }\right)e^{i/\hbar \left(P_{c}-P_{c}^{\prime }\right)\cdot R}e^{i\left(P_{c}^{\prime 2}-P_{c}^{2}\right)t/2\hbar M_{c}}dP_{c}dP_{c}^{\prime }\\ -\frac{1}{2\chi^{2}\left(R,t\right)}\int G\left[\frac{m}{\hbar M_{c}}\left(P_{c}-P_{c}^{\prime }\right)\right]\left(P_{c}-P_{c}^{\prime }\right)f\left(P_{c}\right)f^{*}\left(P_{c}^{\prime }\right)e^{i/\hbar \left(P_{c}-P_{c}^{\prime }\right)\cdot R}e^{i\left(P_{c}^{\prime 2}-P_{c}^{2}\right)t/2\hbar M_{c}}dP_{c}dP_{c}^{\prime }\\ =-\frac{1}{2\chi^{2}\left(R,t\right)}\frac{M}{M_{c}}\int G\left[\frac{m}{\hbar M_{c}}\left(P_{c}-P_{c}^{\prime }\right)\right]\left(P_{c}-P_{c}^{\prime }\right)f\left(P_{c}\right)f^{*}\left(P_{c}^{\prime }\right)e^{i/\hbar \left(P_{c}-P_{c}^{\prime }\right)\cdot R}e^{i\left(P_{c}^{\prime 2}-P_{c}^{2}\right)t/2\hbar M_{c}}dP_{c}dP_{c}^{\prime }\\ +\frac{1}{\chi^{2}\left(R,t\right)}\frac{M}{M_{c}}\int G\left[\frac{m}{\hbar M_{c}}\left(P_{c}-P_{c}^{\prime }\right)\right]P_{c}f\left(P_{c}\right)f^{*}\left(P_{c}^{\prime }\right)e^{i/\hbar \left(P_{c}-P_{c}^{\prime }\right)\cdot R}e^{i\left(P_{c}^{\prime 2}-P_{c}^{2}\right)t/2\hbar M_{c}}dP_{c}dP_{c}^{\prime }\\ =-i\frac{1}{\chi^{2}\left(R,t\right)}\frac{M}{M_{c}}Im\int G\left[\frac{m}{\hbar M_{c}}\left(P_{c}-P_{c}^{\prime }\right)\right]P_{c}f\left(P_{c}\right)f^{*}\left(P_{c}^{\prime }\right)e^{i/\hbar \left(P_{c}-P_{c}^{\prime }\right)\cdot R}e^{i\left(P_{c}^{\prime 2}-P_{c}^{2}\right)t/2\hbar M_{c}}dP_{c}dP_{c}^{\prime }\\ +\frac{1}{\chi^{2}\left(R,t\right)}\frac{M}{M_{c}}\int G\left[\frac{m}{M+m}\left(P_{c}-P_{c}^{\prime }\right)\right]P_{c}f\left(P_{c}\right)f^{*}\left(P_{c}^{\prime }\right)e^{i/\hbar \left(P_{c}-P_{c}^{\prime }\right)\cdot R}e^{i\left(P_{c}^{\prime 2}-P_{c}^{2}\right)t/2\hbar M_{c}}dP_{c}dP_{c}^{\prime }\\ =\frac{1}{\chi^{2}\left(R,t\right)}\frac{M}{M_{c}}Re\int G\left[\frac{m}{\hbar M_{c}}\left(P_{c}-P_{c}^{\prime }\right)\right]P_{c}f\left(P_{c}\right)f^{*}\left(P_{c}^{\prime }\right)e^{i/\hbar \left(P_{c}-P_{c}^{\prime }\right)\cdot R}e^{i\left(P_{c}^{\prime 2}-P_{c}^{2}\right)t/2\hbar M_{c}}dP_{c}dP_{c}^{\prime } \end{array}$$

Then, for the Berry curvature magnetic field we have

$$\begin{array}{l} B\left(R,t\right)=\nabla_{R}\times A\left(R,t\right)=\frac{M}{M_{c}}Re[\nabla_{R}\frac{1}{\chi^{2}\left(R,t\right)}\times \int G\left[\frac{m}{\hbar M_{c}}\left(P_{c}-P_{c}^{\prime }\right)\right]P_{c}f\left(P_{c}\right)f^{*}\left(P_{c}^{\prime }\right)e^{i/\hbar \left(P_{c}-P_{c}^{\prime }\right)\cdot R}e^{i\left(P_{c}^{\prime 2}-P_{c}^{2}\right)t/2\hbar M_{c}}dP_{c}dP_{c}^{\prime }\\ +\frac{1}{\chi^{2}\left(R,t\right)}\nabla_{R}\times \int G\left[\frac{m}{\hbar M_{c}}\left(P_{c}-P_{c}^{\prime }\right)\right]P_{c}f\left(P_{c}\right)f^{*}\left(P_{c}^{\prime }\right)e^{i/\hbar \left(P_{c}-P_{c}^{\prime }\right)\cdot R}e^{i\left(P_{c}^{\prime 2}-P_{c}^{2}\right)t/2\hbar M_{c}}dP_{c}dP_{c}^{\prime }]\\ =\frac{M}{\hbar M_{c}}[-\frac{i}{\chi^{4}\left(R,t\right)}\int f\left(P_{c}\right)f^{*}\left(P_{c}^{\prime }\right)\left(P_{c}-P_{c}^{\prime }\right)e^{i\left(P_{c}-P_{c}^{\prime }\right)\cdot R}G\left[\frac{m}{\hbar M_{c}}\left(P_{c}-P_{c}^{\prime }\right)\right]e^{iP_{c}^{\prime 2}-P_{c}^{2}/2\hbar M_{c}t}dP_{c}dP_{c}^{\prime }\\ \times Re\int G\left[\frac{m}{\hbar M_{c}}\left(P_{c}-P_{c}^{\prime }\right)\right]P_{c}f\left(P_{c}\right)f^{*}\left(P_{c}^{\prime }\right)e^{i/\hbar \left(P_{c}-P_{c}^{\prime }\right)\cdot R}e^{i\left(P_{c}^{\prime 2}-P_{c}^{2}\right)t/2\hbar M_{c}}dP_{c}dP_{c}^{\prime }\\ +\frac{1}{\chi^{2}\left(R,t\right)}Rei\int G\left[\frac{m}{\hbar M_{c}}\left(P_{c}-P_{c}^{\prime }\right)\right]P_{c}\times P_{c}^{\prime }f\left(P_{c}\right)f^{*}\left(P_{c}^{\prime }\right)e^{i/\hbar \left(P_{c}-P_{c}^{\prime }\right)\cdot R}e^{i\left(P_{c}^{\prime 2}-P_{c}^{2}\right)t/2\hbar M_{c}}dP_{c}dP_{c}^{\prime }]\\ =\frac{M}{\hbar M_{c}}[\frac{1}{\chi^{4}\left(R,t\right)}Im\int f\left(P_{c}\right)f^{*}\left(P_{c}^{\prime }\right)\left(P_{c}-P_{c}^{\prime }\right)e^{i\left(P_{c}-P_{c}^{\prime }\right)\cdot R}G\left[\frac{m}{\hbar M_{c}}\left(P_{c}-P_{c}^{\prime }\right)\right]e^{iP_{c}^{\prime 2}-P_{c}^{2}/2\hbar M_{c}t}dP_{c}dP_{c}^{\prime }\\ \times Re\int G\left[\frac{m}{\hbar M_{c}}\left(P_{c}-P_{c}^{\prime }\right)\right]P_{c}f\left(P_{c}\right)f^{*}\left(P_{c}^{\prime }\right)e^{i/\hbar \left(P_{c}-P_{c}^{\prime }\right)\cdot R}e^{i\left(P_{c}^{\prime 2}-P_{c}^{2}\right)t/2\hbar M_{c}}dP_{c}dP_{c}^{\prime }\\ -\frac{1}{\chi^{2}\left(R,t\right)}Im\int G\left[\frac{m}{\hbar M_{c}}\left(P_{c}-P_{c}^{\prime }\right)\right]P_{c}\times P_{c}^{\prime }f\left(P_{c}\right)f^{*}\left(P_{c}^{\prime }\right)e^{i/\hbar \left(P_{c}-P_{c}^{\prime }\right)\cdot R}e^{i\left(P_{c}^{\prime 2}-P_{c}^{2}\right)t/2\hbar M_{c}}dP_{c}dP_{c}^{\prime }]\\ =\frac{M}{\hbar M_{c}}Im[\frac{1}{\chi^{4}\left(R,t\right)}\int f\left(P_{c}\right)f^{*}\left(P_{c}^{\prime }\right)\left(P_{c}-P_{c}^{\prime }\right)e^{i\left(P_{c}-P_{c}^{\prime }\right)\cdot R}G\left[\frac{m}{\hbar M_{c}}\left(P_{c}-P_{c}^{\prime }\right)\right]e^{iP_{c}^{\prime 2}-P_{c}^{2}/2\hbar M_{c}t}dP_{c}dP_{c}^{\prime }\\ \times \int G\left[\frac{m}{\hbar M_{c}}\left(P_{c}-P_{c}^{\prime }\right);\frac{mM}{M_{c}}\right]P_{c}f\left(P_{c}\right)f^{*}\left(P_{c}^{\prime }\right)e^{i/\hbar \left(P_{c}-P_{c}^{\prime }\right)\cdot R}e^{i\left(P_{c}^{\prime 2}-P_{c}^{2}\right)t/2\hbar M_{c}}dP_{c}dP_{c}^{\prime }\\ -\frac{1}{\chi^{2}\left(R,t\right)}\int G\left[\frac{m}{\hbar M_{c}}\left(P_{c}-P_{c}^{\prime }\right)\right]P_{c}\times P_{c}^{\prime }f\left(P_{c}\right)f^{*}\left(P_{c}^{\prime }\right)e^{i/\hbar \left(P_{c}-P_{c}^{\prime }\right)\cdot R}e^{i\left(P_{c}^{\prime 2}-P_{c}^{2}\right)t/2\hbar M_{c}}dP_{c}dP_{c}^{\prime }]\\ =\frac{M}{\hbar M_{c}}Im[\frac{1}{\chi^{4}\left(R,t\right)}\int P_{c}f\left(P_{c}\right)f^{*}\left(P_{c}^{\prime }\right)e^{i\left(P_{c}-P_{c}^{\prime }\right)\cdot R}G\left[\frac{m}{\hbar M_{c}}\left(P_{c}-P_{c}^{\prime }\right)\right]e^{iP_{c}^{\prime 2}-P_{c}^{2}/2\hbar M_{c}t}dP_{c}dP_{c}^{\prime }\\ \times \int P_{c}^{\prime }f\left(P_{c}\right)f^{*}\left(P_{c}^{\prime }\right)e^{i/\hbar \left(P_{c}-P_{c}^{\prime }\right)\cdot R}G\left[\frac{m}{\hbar M_{c}}\left(P_{c}-P_{c}^{\prime }\right)\right]e^{i\left(P_{c}^{\prime 2}-P_{c}^{2}\right)t/2\hbar M_{c}}dP_{c}dP_{c}^{\prime }\\ -\frac{1}{\chi^{2}\left(R,t\right)}\int P_{c}\times P_{c}^{\prime }f\left(P_{c}\right)f^{*}\left(P_{c}^{\prime }\right)e^{i\left(P_{c}-P_{c}^{\prime }\right)\cdot R}G\left[\frac{m}{\hbar M_{c}}\left(P_{c}-P_{c}^{\prime }\right)\right]e^{i\left(P_{c}^{\prime 2}-P_{c}^{2}\right)t/2\hbar M_{c}}dP_{c}dP_{c}^{\prime }] \end{array}$$

With the expression after the last equality sign, we conclude the proof of eq 67.
